# Retrospective Analysis of the Epidemiological and Clinical Characteristics of Acute Coronary Syndrome in a Tertiary Hospital Located at High Altitude

**DOI:** 10.3390/jcm14176232

**Published:** 2025-09-03

**Authors:** Vladimir E. Ullauri-Solórzano, Christian Humberto Fierro Renoy, Juan Carlos Gaibor Barba, Diana Moreira-Vera, Henrry Oswaldo Jaramillo Prado, Ana Gabriela Finke Barriga, Víctor Arias Adriano, Francisco Xavier Castro Vega, Ana Cristina Chiliquinga, Kateherine Fonte Melendres, Luis Felipe Varela Pólit, Estefanía Chediak Pérez, Paulina Elizabeth Arteaga Yépez, Luis Alberto Paucar Rojas, Juan S. Izquierdo-Condoy, Jorge Vasconez-Gonzalez, Esteban Ortiz-Prado

**Affiliations:** 1 Hospital Metropolitano de Quito, Quito 170521, Ecuador; 2 One Health Research Group, Universidad de las Americas, Quito 170137, Ecuador

**Keywords:** acute coronary syndrome, STEMI, NSTEMI, cardiovascular diseases, hospital management, high altitude

## Abstract

**Background:** Cardiovascular diseases (CVDs) are the leading global cause of death, responsible for 17.9 million deaths annually (32%) as of 2019. Acute coronary syndrome (ACS) significantly contributes to this burden, particularly in low- and middle-income countries. In Latin America, ACS accounts for 35% of deaths. In Ecuador, 36,058 deaths were reported between 2019 and 2021, underscoring its significant public health impact. **Objectives:** This study aimed to determine the epidemiological, clinical, and hospital management characteristics of ACS patients admitted to the Metropolitan Hospital of Quito, located at high altitude, from January 2021 to October 2023. **Methodology:** A retrospective observational study analyzed anonymized medical records of 133 ACS patients treated at a tertiary care hospital in Quito. **Results:** Among 133 ACS patients, 72.9% were male, with a mean age of 68.9 ± 13.9 years. Overweight was prevalent, with a mean BMI of 26.6 ± 3.0 kg/m^2^. Hypertension (51.9%) and type 2 diabetes mellitus (26.3%) were common comorbidities. ST-segment elevation myocardial infarction (STEMI) was the most frequent ACS type (48.9%), followed by non-ST-segment elevation myocardial infarction (NSTEMI) (33.8%). Most patients (72.2%) underwent stent placement. Mortality was low (2.3%) and significantly associated with the presence of immunologic diseases (*p* = 0.015), TIMI risk score Grade IV (*p* = 0.009), and cardiac arrest on admission (*p* < 0.001). **Conclusions:** This study provides critical insights into the epidemiology and management of ACS in a high-altitude urban setting, emphasizing the importance of timely diagnosis and evidence-based therapies in improving patient outcomes.

## 1. Introduction

Cardiovascular diseases (CVDs) are the leading cause of death and disability worldwide. According to the World Health Organization (WHO), approximately 17.9 million deaths were attributed to CVDs in 2019, accounting for 32% of all global deaths [1]. Among these, ischemic heart disease (IHD), also known as coronary artery disease, and atherosclerotic cardiovascular disease represent significant contributors to morbidity and mortality [2].

Acute coronary syndrome (ACS) encompasses a spectrum of clinical conditions characterized by acute myocardial ischemia, including ST-segment elevation myocardial infarction (STEMI), non-ST-segment elevation myocardial infarction (NSTEMI), and unstable angina [3,4]. Globally, ischemic heart disease is responsible for approximately 12% of disability-adjusted life years (DALYs) lost annually. In Europe alone, IHD causes more than 860,000 deaths among men and approximately 880,000 deaths among women each year [2,5]. Moreover, ACS accounts for one-third of all deaths in individuals over 35 years of age, with a disproportionately high burden in low- and middle-income countries. These countries experience nearly 7 million deaths and 129 million DALYs annually due to ACS [6].

In many low- and middle-income regions, rapid economic growth and lifestyle changes over the past decades have increased the prevalence of cardiovascular risk factors, contributing to rising mortality rates. Latin America is one such region, with a significant burden of cardiovascular risk factors, including overweight, dyslipidemia, diabetes mellitus, and infectious diseases like Chagas disease and rheumatic fever [7,8]. The WHO estimates that ACS accounts for 35% of all deaths in Latin America, and projections suggest that over the next four decades, deaths from cardiovascular diseases in the region will rise by more than 200% [6,8].

In Ecuador, between 2019 and 2021, 36,058 deaths from acute myocardial infarction were reported. The provinces in the coastal region exhibited the highest mortality rates in the country. During this period, the mortality rate increased by 79.6%, leading to a significant public health burden, with 339,067.6 years of life lost prematurely [9].

Traditional cardiovascular risk factors like smoking, obesity, and diabetes are well-established contributors to atherosclerosis. In contrast, non-traditional factors such as hypoxia and high-altitude exposure are less studied [2]. Although acclimatization enables physiological adaptation, it may become pathological in individuals with comorbidities, as the cardiovascular system compensates for reduced oxygen by increasing workload [10]. High-altitude hypoxia triggers increased cardiac output, coronary vasodilation, and pulmonary vasoconstriction [10]. After acclimatization, cardiac output normalizes, but elevated sympathetic tone and tissue hypoxia often persist. Heart rate remains high, while stroke volume decreases [11].

Although healthy individuals generally tolerate high-altitude exposure without an increased risk of myocardial ischemia, individuals with a history of cardiovascular disease may experience adverse effects. High-altitude environments may unpredictably precipitate acute coronary events or exacerbate anginal symptoms [11].

The aim of this study is to determine the epidemiological, clinical, and hospital management characteristics of patients admitted with acute coronary syndrome, with or without ST-segment elevation, at the Metropolitan Hospital of Quito, located at high altitude from January 2021 to October 2023.

## 2. Materials and Methods

### 2.1. Study Desing

A retrospective observational study was conducted to assess clinical outcomes and associated factors among patients with acute coronary syndrome. The study was based on the analysis of anonymized medical records from the Metropolitan Hospital of Quito, a leading third-level care facility in Quito, Ecuador.

### 2.2. Setting

The study was conducted at the Metropolitan Hospital of Quito, a specialty care center serving a predominantly private sector population in Quito, Ecuador. Quito, the capital city of Ecuador, is located at approximately 2800 m above sea level and has a population of approximately 2.7 million.

### 2.3. Sample

Non-probabilistic consecutive sampling was employed to collect data from medical records, ensuring the inclusion of all eligible patients treated for acute coronary syndrome during the study period. This approach allowed for the comprehensive capture of cases treated in the cardiology service from January 2021 to December 2023.

### 2.4. Inclusion and Exclusion Criteria

The inclusion criteria comprised patients aged ≥18 years with a confirmed diagnosis of acute coronary syndrome (ACS), with or without ST-segment elevation, who received care at the Cardiology Department of Hospital Metropolitano de Quito between January 2021 and December 2023. This included patients referred from other healthcare facilities, as well as those previously hospitalized for non-ACS-related conditions who later presented with cardiovascular symptoms.

The exclusion criteria encompassed patients under 18 years of age, cases with insufficient clinical data, medical records outside the study period, and patients diagnosed with cardiac conditions other than ACS.

### 2.5. Sample and Data Collection

A total of 138 clinical records with a diagnosis of acute coronary syndrome were identified from the cardiology service database. After applying the inclusion and exclusion criteria, 5 clinical records were excluded due to insufficient data, resulting in a final sample size of 133 clinical records. An anonymized database was constructed, which captured demographic variables (e.g., sex, age at diagnosis, nationality, residence, and ethnicity), anthropometric measurements, and clinical variables, including comorbidities, forms of presentation, symptoms, types of acute coronary syndrome, variables characterizing hospital admission, and characteristics of hospital management of patients, including treatment and clinical outcomes. To ensure data accuracy and integrity, two independent members of the research team conducted a double-check process, cross-verifying the entered data against the original medical records. All data were anonymized and recorded in a secure Excel spreadsheet. No personally identifiable information was collected, and access to the dataset was strictly limited to authorized members of the research team.

### 2.6. Statistical Analysis

Descriptive statistics were used to summarize the variables, with frequencies and percentages for categorical variables and measures of central tendency (mean and standard deviation) for numerical variables. Normality was assessed using the Shapiro–Wilk test. Chi-square and Student’s *t* tests were applied to assess associations and mean differences with acute coronary syndrome outcomes, respectively, with a significance level of *p* < 0.05. Data analysis was performed using IBM SPSS Statistics for Windows, version 29.0 (IBM Corporation, Chicago, IL, USA).

### 2.7. Ethical Statement

The study adhered to the ethical principles outlined in the Declaration of Helsinki and was approved by the Ethics Committee for Research in Human Beings of the University of the Americas (CEISH-UDLA) under protocol code 2024-OBS-020. The research protocol, informed consent form, and database format were reviewed and approved. Given the retrospective and observational nature of the study, the ethics committee approved a waiver of informed consent, ensuring compliance with ethical and methodological standards. Patient confidentiality was rigorously protected through data anonymization, and all procedures conformed to the applicable ethical and legal regulations for research in Ecuador. Supporting documentation and evaluation forms are archived at CEISH-UDLA.

## 3. Results

### 3.1. Demographic Characteristics and Personal History

This study included 133 patients diagnosed with acute coronary syndrome (ACS), the majority being male (72.9%) and of mestizo ethnicity (94.7%), with a mean age of 68.9 ± 13.9 years (Table 1). When stratified by sex and in-hospital outcome (alive vs. deceased), 2.1% of men and 2.8% of women died during hospitalization. Baseline characteristics showed a higher prevalence of cardiovascular risk factors among men, including smoking (32.0%) and dyslipidemia (16.5%), while women had slightly higher rates of diabetes (33.3%) and immunologic disease (8.3%). ST-segment elevation myocardial infarction (STEMI) was the most common presentation overall, affecting 54.6% of men and 36.1% of women. Detailed demographic and clinical characteristics, disaggregated by sex and survival outcome, are shown in Table 2.

Anthropometric and pathological data further highlight the comorbidity burden among patients with ACS. The mean body mass index (BMI) was 26.6 ± 3.0 kg/m^2^, indicating a high prevalence of being overweight. Smoking was reported by 25.6% of the total population. Regarding clinical history, 23.3% of patients had a previous myocardial infarction, 51.9% had hypertension, and 26.3% had type 2 diabetes mellitus. Other conditions such as heart failure, atrial fibrillation, and immunologic diseases were less common, although the latter showed a statistically significant association with in-hospital mortality (*p* = 0.015) (Table 1).

### 3.2. Characteristics of Acute Coronary Syndrome

ST-segment elevation myocardial infarction (STEMI) was the most frequent ACS type, accounting for 48.9% of cases, followed by non-ST-segment elevation myocardial infarction (NSTEMI) at 33.8% and unstable angina at 17.3%. Among symptomatic patients, precordial pain was the predominant symptom (85.7%). According to the TIMI Risk Score, grade III was assigned to 43.6% of cases, and 40.6% of patients exhibited low risk by GRACE Risk Category. Among coronary arteries, the Left Anterior Descending (LAD) artery was the most affected (37.6%), followed by the Right Coronary Artery (RCA) artery (20.3%). Management primarily involved stent implantation (72.2%) (Table 3).

### 3.3. Admission and Hospital Management

Upon hospital admission, patients had a mean heart rate of 78.0 ± 17.0 bpm, a mean systolic blood pressure of 129.1 ± 25.2 mmHg, and a diastolic blood pressure of 79.3 ± 16.1 mmHg. The most common electrocardiographic finding was ST-segment elevation, observed in 45.9% of patients. Arrhythmias were rare, with atrial fibrillation occurring in 2.3% of cases and ventricular fibrillation in 1.4%. Additionally, 12.8% of patients presented with mitral regurgitation (Table 3). Regarding wall motion abnormalities, 86 patients (64.7%) exhibited abnormal motion, of whom 55.8% showed evidence of hypokinesia, suggesting underlying structural cardiac alterations. In terms of intervention times, patients who survived had similar door-to-ECG and door-to-balloon times, with a mean of 10.8 ± 6.0 min and 73.5 ± 128.6 min, respectively. However, deceased patients showed shorter times in both metrics, with a mean of 10.0 min for ECG and 10.0 min for door-to-balloon time (*p* < 0.001) (Table 4).

The mean hospital stay was 5 ± 23 days. NYHA class I was the most frequently observed functional classification, reported in 36.8% of patients. In-Hospital pharmacological management primarily involved the use of statins (97.7%), acetylsalicylic acid (97.7%), and anticoagulants (85.7%), while enoxaparin was prescribed in only 68.4% of anticoagulant use cases.

At discharge, approximately 52.6% was classified as NYHA class I, and 42.9% NYHA class II. In addition, 97.7% of patients received statins, and acetylsalicylic acid, followed by Clopidogrel or Ticagrelor in 76.7% (Table 5).

### 3.4. Factors Related to Mortality

In this cohort, the overall in-hospital mortality was 2.3%. Demographic variables did not significantly differ between survivors and non-survivors. However, a significantly higher prevalence of immunologic diseases was observed among deceased patients (16.7%; *p* < 0.015) (Table 1).

Regarding clinical characteristics of ACS, a Grade IV score on the TIMI risk scale was significantly associated with mortality (8.3%; *p* = 0.009). Additionally, the femoral artery was more frequently used as the coronary access route in patients who died (16.7%; *p* = 0.002), suggesting a potential procedural association with worse outcomes (Table 3).

Upon hospital admission, a higher percentage of deaths occurred in patients without ST-segment elevation (2.8%; *p* = 0.892). However, critical factors significantly associated with mortality included lower systolic blood pressure in deceased patients (mean 93.3 ± 37.8 mmHg) compared to survivors (mean 129.6 ± 23.8 mmHg; *p* < 0.001), as well as reduced pulmonary artery systolic pressure (mean 0.0 ± 0.0 mmHg; *p* < 0.001). Additionally, cardiac arrest at presentation was observed in all patients who died (100.0%; *p* < 0.001), underscoring its prognostic relevance in acute clinical settings (Table 4).

## 4. Discussion

This study highlights the epidemiological and clinical characteristics of ACS in a high-altitude urban setting in Quito, Ecuador, emphasizing the influence of geographic and physiological factors on cardiovascular outcomes. The management of ACS at elevations such as Quito’s 2800 m above sea level presents unique challenges that remain underexplored in the global literature, particularly within Latin American contexts.

The demographic profile observed in this cohort aligns with global epidemiological patterns, where older males constituted the majority of ACS cases (72.9%). The high prevalence of overweight individuals (mean BMI: 26.6 kg/m^2^) reinforces the growing burden of obesity as a modifiable risk factor for cardiovascular disease in Ecuador. Moreover, hypertension (51.9%) and type 2 diabetes mellitus (26.3%) were the most prevalent comorbidities—consistent with both regional and international data on ACS risk factors.

As usual, STEMI was the predominant ACS subtype (48.9%), followed by NSTEMI (33.9%) and unstable angina (17.3%). This distribution may be influenced by chronic high-altitude exposure, which has been associated with several physiological adaptations and maladaptations that impact ischemic heart disease. These include hypobaric hypoxia—contributing to elevated blood viscosity and systemic vascular resistance [12,13,14], as well as increased sympathetic tone, right ventricular strain, respiratory alkalosis, altered potassium transport, endothelial dysfunction, and a prothrombotic state characterized by enhanced platelet aggregation and hypercoagulability [10]. These high-altitude stressors may alter the clinical presentation and therapeutic response of ACS and may explain the attenuated progression of non-culprit lesions observed in patients residing at moderate altitudes [15,16]. Context-specific research conducted in Quito could yield critical insights into these pathophysiological mechanisms. Our data contradicts this appreciation.

Supporting this perspective, a study of 768 patients with ACS found that individuals residing at higher altitudes exhibited significantly higher rates of hyperlipidemia, pre-existing coronary artery disease, and diabetes mellitus. Moreover, ACS tended to occur at younger ages in these populations and was associated with a higher incidence of stroke and reduced left ventricular ejection fraction. Laboratory parameters, including hemoglobin, hematocrit, and white blood cell counts, were also significantly elevated at high altitude, further underscoring the physiological burden imposed by chronic hypoxia [17].

Clinical variables in this cohort revealed that surviving patients had a significantly lower mean heart rate (77.4 ± 15.7 bpm; *p* < 0.054), while systolic blood pressure was significantly higher among survivors (129.6 ± 23.8 mmHg; *p* < 0.011). Precordial pain was the most frequently reported symptom (85.7%). Coronary angiography identified the left anterior descending (LAD) artery as the most commonly affected vessel (37.6%), followed by the right coronary artery (RCA; 52.2%). Pharmacologic management adhered to standard care guidelines, with 97.7% of patients receiving statins and acetylsalicylic acid, and 81.2% receiving thienopyridines (clopidogrel or ticagrelor). Importantly, the absence of statins, beta blockers, aspirin, and thienopyridines was significantly associated with mortality, highlighting the critical role of evidence-based pharmacotherapy in reducing adverse outcomes.

Anticoagulant agents administered included apixaban, enoxaparin, heparin, and rivaroxaban. Recent research has emphasized the importance of pharmacogenomics in modulating drug efficacy and safety, particularly in cardiovascular pharmacotherapy. For example, CYP3A4 polymorphisms have been linked to diminished rivaroxaban activity, while the ABCG2 421C>A variant results in increased apixaban plasma concentrations [18]. In heparin-treated patients, genetic variants may induce resistance or elevate the risk of heparin-induced thrombocytopenia [18], suggesting a role for personalized medicine in ACS treatment protocols.

The in-hospital mortality rate in this study was low (2.3%), likely reflecting the high-quality care provided at Hospital Metropolitano. Nevertheless, several factors were significantly associated with mortality, including the use of femoral access for coronary intervention, hypotension on admission, cardiac arrest at presentation, and the lack of pharmacological therapy. These findings are consistent with international data emphasizing the need for early intervention and strict adherence to clinical guidelines [19,20,21,22].

Risk stratification based on modifiable clinical characteristics remains vital for implementing targeted interventions. In this context, inflammatory indices have gained attention as non-invasive and readily accessible prognostic tools in ACS [23]. Among these, the systemic immune-inflammation index (SII) has emerged as a novel marker capable of quantifying the intensity of systemic inflammatory responses following coronary events. Elevated SII values have been independently associated with adverse cardiovascular outcomes in patients undergoing primary coronary angiography [24]. In addition, Trimarchi et al. [23] evaluated the prognostic value of the advanced lung cancer inflammation index (ALI) in patients with ST-segment elevation myocardial infarction undergoing primary percutaneous coronary intervention. Their study demonstrated that ALI is an independent predictor of all-cause mortality in this population. Notably, patients with ALI values ≤ 10 had a 2.3-fold higher risk of mortality compared to those with ALI values > 10, suggesting its potential utility in clinical risk stratification [23].

Additionally, the study sheds light on the gaps in high-altitude ACS management in Latin America. While regions like the French Alps and the Himalayas have developed effective emergency networks and adaptive strategies [25], similar frameworks are underdeveloped in Ecuador. Region-specific protocols tailored to high-altitude settings could significantly improve outcomes and provide equitable care across diverse healthcare settings.

## 5. Limitations

This study has several notable limitations. First, its retrospective observational design depends on the accuracy and completeness of medical records, which could introduce information bias or restrict the availability of relevant data. Second, the study was conducted at a single tertiary care hospital serving primarily a private-sector population in Quito, Ecuador—a high-altitude urban setting. This context may not reflect the healthcare experiences or outcomes of patients from public institutions, rural areas, or differing socioeconomic backgrounds, thereby limiting the external validity of the findings. A key limitation is the absence of a comparison group from low-altitude regions. Due to logistical constraints and limited access, the research team was unable to recruit or analyze data from patients residing at lower altitudes. This lack of comparative analysis restricts the study’s capacity to evaluate how altitude-specific physiological and clinical factors may influence the presentation and outcomes of ACS. Additionally, the use of non-probability consecutive sampling may not fully represent the spectrum of acute coronary syndrome cases in the region. Finally, the relatively small sample size—combined with the low observed mortality (only one recorded death)—limits the statistical power to detect robust associations between clinical variables and outcomes. Addressing these limitations in future studies could provide a more comprehensive understanding of acute coronary syndrome in diverse populations.

## 6. Conclusions

This study provides valuable insights into the characteristics and management of ACS (acute coronary syndrome) in a high-altitude urban environment, addressing an important knowledge gap in the Latin American context. Recognizing that both physiological and environmental factors associated with altitude can influence the clinical presentation, diagnosis, and treatment of this condition is of utmost importance. Therefore, future research should include larger samples that encompass both residents of high-altitude and low-altitude regions, as well as diverse healthcare settings in both urban and rural environments. This would allow for a better understanding of the similarities and differences in cases, diagnoses, and treatments. Additionally, the development of longitudinal studies is necessary to comprehensively investigate the dynamics of ACS in high-altitude environments.

Understanding the physiological adaptations and unique management strategies for high-altitude environments will be essential for developing comprehensive cardiovascular care frameworks for Ecuador and similar regions. In this way, it would be possible to create a care model tailored to the specific needs of each population.

## Figures and Tables

**Table 1:** Demographic and medical history of patients with acute coronary syndrome by outcome.

		Outcome						*p*-Value
		Total		Alive		Deceased		
		n	%	n	%	n	%	
Sex	Male	97	72.9	95	97.9	2	2.1	0.805
	Female	36	27.1	35	97.2	1	2.8	
Residence	Ecuador	130	97.7	127	97.7	3	2.3	0.790
	Foreign	3	2.3	3	100.0	0	0.0	
Age (years)	Mean (±SD)	68.9	13.9	68.6	14.1	68.3	4.2	0.971 *
Ethnicity	White	7	5.3	7	100.0	0	0.0	0.680
	Mestizo	126	94.7	123	97.6	3	2.4	
BMI (kg/m^2^)	Mean (±SD)	26.6	3.4	27.4	3.1	23.6	1.6	0.051 *
Alcohol Consumption	Yes	10	7.5	10	100.0	0	0.0	0.617
Smoking	Yes	34	25.6	34	100.0	0	0.0	0.305
Hospitalization for Same Cause (30 Days)	Yes	4	3	4	100.0	0	0.0	0.758
Physical Activity (3 times a week)	Yes	35	26.3	35	100.0	0	0.0	0.295
Medical History								
History of Cardiac Surgery	Yes	3	2.3	3	100.0	0	0.0	0.790
Type of Surgery	None	130	97.7	127	97.7	3	2.3	0.965
	Valvular	1	0.8	1	100.0	0	0.0	
	Coronary	2	1.5	2	100.0	0	0.0	
Myocardial Infarction (MI)	Yes	31	23.3	29	93.5	2	6.5	0.072
Hypertension	Yes	69	51.9	67	97.1	2	2.9	0.604
Type 2 Diabetes	Yes	35	26.3	33	94.3	2	5.7	0.108
Dyslipidemia	Yes	25	18.8	24	96.0	1	4.0	0.515
History of PE, DVT, or Stroke	Yes	10	7.5	10	100.0	0	0.0	0.617
Obesity	Yes	30	22.6	30	100.0	0	0.0	0.344
Chronic Kidney Disease	Yes	3	2.3	3	100.0	0	0.0	0.790
Thyroid Disease	Yes	17	12.8	17	100.0	0	0.0	0.502
Chagas Serology	Yes	1	0.8	1	100.0	0	0.0	0.879
Sleep Apnea	Yes	3	2.3	3	100.0	0	0.0	0.79
Use of CPAP	Yes	3	2.3	3	100.0	0	0.0	0.79
Immunologic Diseases	Yes	6	4.5	5	83.3	1	16.7	**0.015**
Heart Failure	Yes	2	1.5	2	100.0	0	0.0	0.829
Arrhythmia	Yes	5	1.5	5	100.0	0	0.0	0.729
Pacemaker Use	Yes	2	1.5	2	100.0	0	0.0	0.829
Prior Use of Defibrillator	No	133	100.0	130	97.7	3	2.3	N/A

SD: Standard deviation, BMI: body mass index, MI: myocardial infarction, PE: pulmonary embolism, DVT: deep vein thrombosis, CPAP: continuous positive airway pressure. * *p* values calculated from Student’s *t*-test. Values in **bold** indicate statistically significant differences at *p* < 0.05.

**Table 2:** Clinical characteristics, ACS presentation, and in-hospital outcomes stratified by sex and survival status.

Variable	Men (n = 97)	Alive (n = 95)	Deceased (n = 2)	Women (n = 36)	Alive (n = 35)	Deceased (n = 1)
Age (years, mean ± SD)	68.0 ± 14.2	68.1 ± 14.3	70.0 ± 4.2	70.4 ± 12.9	70.5 ± 13.1	65.0 ± 0.0
BMI (kg/m^2^, mean ± SD)	26.9 ± 3.1	26.9 ± 3.1	24.7 ± 1.4	26.1 ± 3.8	26.2 ± 3.9	22.3 ± 0.0
Hypertension (%)	53.6 (52/97)	54.7 (52/95)	0.0 (0/2)	47.2 (17/36)	48.6 (17/35)	0.0 (0/1)
Diabetes (%)	22.7 (22/97)	21.1 (20/95)	100.0 (2/2)	33.3 (12/36)	34.3 (12/35)	0.0 (0/1)
Dyslipidemia (%)	16.5 (16/97)	15.8 (15/95)	50.0 (1/2)	25.0 (9/36)	25.7 (9/35)	0.0 (0/1)
Smoking (%)	32.0 (31/97)	32.6 (31/95)	0.0 (0/2)	13.9 (5/36)	14.3 (5/35)	0.0 (0/1)
Immunologic Disease (%)	3.1 (3/97)	2.1 (2/95)	50.0 (1/2)	8.3 (3/36)	8.6 (3/35)	0.0 (0/1)
ACS Type: STEMI (%)	54.6 (53/97)	54.7 (52/95)	50.0 (1/2)	36.1 (13/36)	37.1 (13/35)	0.0 (0/1)
TIMI Grade IV (%)	10.3 (10/97)	9.5 (9/95)	50.0 (1/2)	5.6 (2/36)	5.7 (2/35)	0.0 (0/1)
Femoral Access (%)	8.2 (8/97)	7.4 (7/95)	50.0 (1/2)	11.1 (4/36)	8.6 (3/35)	100.0 (1/1)
Stent Placement (%)	76.3 (74/97)	76.8 (73/95)	50.0 (1/2)	63.9 (23/36)	65.7 (23/35)	0.0 (0/1)
Heart Rate (bpm, mean ± SD)	77.1 ± 16.5	76.7 ± 15.9	94.5 ± 35.4	80.0 ± 18.1	79.8 ± 18.4	85.0 ± 0.0
Systolic BP (mmHg, mean ± SD)	130.7 ± 24.3	131.2 ± 24.0	85.0 ± 49.5	125.8 ± 26.7	127.8 ± 25.4	50.0 ± 0.0
Cardiac Arrest (%)	2.1 (2/97)	0.0 (0/95)	100.0 (2/2)	2.8 (1/36)	0.0 (0/35)	100.0 (1/1)
LVEF (%, mean ± SD)	53.2 ± 20.1	54.0 ± 19.6	0.0 ± 0.0	48.5 ± 22.8	49.9 ± 22.3	0.0 ± 0.0
Statins (%)	98.0 (95/97)	100.0 (95/95)	0.0 (0/2)	97.2 (35/36)	100.0 (35/35)	0.0 (0/1)
Beta-blockers (%)	58.8 (57/97)	60.0 (57/95)	0.0 (0/2)	50.0 (18/36)	51.4 (18/35)	0.0 (0/1)
ASA (%)	97.9 (95/97)	100.0 (95/95)	0.0 (0/2)	97.2 (35/36)	100.0 (35/35)	0.0 (0/1)
Clopidogrel/Ticagrelor (%)	83.5 (81/97)	85.3 (81/95)	0.0 (0/2)	75.0 (27/36)	77.1 (27/35)	0.0 (0/1)
Mortality (%)	2.1 (2/97)	-	-	2.8 (1/36)	-	-

SD: Standard deviation; BMI: body mass index; ACS: acute coronary syndrome; STEMI: ST-segment elevation myocardial infarction; TIMI: thrombolysis in myocardial infarction; bpm: beats per minute; BP: blood pressure; LVEF: left ventricular ejection fraction; ASA: acetylsalicylic acid.

**Table 3:** Clinical and procedural characteristics of patients with ACS by outcome.

		Outcome						*p*-Value
		Total		Alive		Deceased		
		n	%	n	%	n	%	
Type of ACS	NSTEMI	45	33.8	45	100.0	0	0.0	0.063
	STEMI	65	48.9	64	98.5	1	1.5	
	Unstable Angina	23	17.3	21	91.3	2	8.7	
Type of ACS Symptom	Precordial Pain	114	85.7	111	97.4	3	2.6	0.972
	Dyspnea	7	5.3	7	100.0	0	0.0	
	Sincope	3	2.3	3	100.0	0	0.0	
	Palpitaciones	2	1.5	2	100.0	0	0.0	
	Otros	7	5.3	7	100.0	0	0.0	
GRACE Risk Category	High	42	31.6	41	97.6	1	2.4	0.504
	Intermediate	37	27.8	37	100.0	0	0.0	
	Low	54	40.6	52	96.3	2	3.7	
TIMI Risk Score	Not Applicable	12	9	10	83.3	2	16.7	**0.009**
	Grade I	21	15.8	21	100.0	0	0.0	
	Grade II	28	21.1	28	100.0	0	0.0	
	Grade III	58	43.6	58	100.0	0	0.0	
	Grade IV	12	9	11	91.7	1	8.3	
Coronary Angiography Performed	Yes	133	100.0	130	97.7	3	2.3	
Coronary Access Route	Radial	121	91	120	99.2	1	0.8	**0.002**
	Femoral	12	9	10	83.3	2	16.7	
Affected Coronary Artery	None	24	18	23	95.8	1	4.2	0.305
	Left Anterior Descending (LAD)	50	37.6	50	100.0	0	0.0	
	Right Coronary Artery (RCA)	27	20.3	27	100.0	0	0.0	
	Circumflex Artery (Cx)	15	11.3	14	93.3	1	6.7	
	Other	2	1.5	2	100.0	0	0.0	
	LAD + RCA	4	3	4	100.0	0	0.0	
	LAD + Cx	7	5.3	6	85.7	1	14.3	
	RCA + Cx	4	3	4	100	0	0	
Stent Placement	No	37	27.8	35	94.6	2	5.4	0.129
	Yes	96	72.2	95	99.0	1	1.0	
Number of Stents Placed	0	37	27.8	35	94.6	2	5.4	0.377
	1	72	54.1	72	100.0	0	0.0	
	2	19	14.3	18	94.7	1	5.3	
	3	3	2.3	3	100.0	0	0.0	
	4	2	1.5	2	100.0	0	0.0	

ACS: Acute coronary syndrome; NSTEMI: non-ST-segment elevation myocardial infarction; STEMI: ST-segment elevation myocardial infarction; GRACE: Global Registry of Acute Coronary Events; TIMI: thrombolysis in myocardial infarction; LAD: left anterior descending (coronary artery); RCA: right coronary artery; Cx: circumflex artery. Values in **bold** indicate statistically significant differences at *p* < 0.05.

**Table 4:** Admission, laboratory, and in-hospital clinical characteristics of patients with ACS by outcome.

		Outcome						*p*-Value
		Total		Alive		Deceased		
		n	%	n	%	n	%	
Heart Rate (bpm)	Mean (±SD)	78	17	77.4	16.7	97	39.1	0.054
Systolic Blood Pressure (mmHg)	Mean (±SD)	129	25	129.6	23.8	93.3	37.8	0.011 *
Diastolic Blood Pressure (mmHg)	Mean (±SD)	79	16	79.1	15.5	63.3	20.8	0.086
Creatinine (mg/dL)	Mean (±SD)	1.1	0.4	1.1	0.4	0.8	0.7	0.526 *
Baseline Glomerular Filtration Rate (eGFR)	Mean (±SD)	75.13	22.08	75.21	22.17	71.67	21.13	0.785 *
Hemoglobin (g/dL)	Mean (±SD)	15.3	4.1	15.3	4.1	12.3	3.6	0.223 *
Hematocrit (%)	Mean (±SD)	45.1	7.6	45.3	7.6	34.8	10.2	0.215 *
Door-to-ECG time (minutes)	Mean (±SD)	10.8	6.0	10.8	6.1	10.0	0.0	0.166
Door-to-balloon time (minutes)	Mean (±SD)	71.9	127.4	73.5	128.6	10.0	1.0	<0.001 *
Cardiac Arrest on Admission	No	130	97.7	130	100.0	0	0.0	<0.001 *
	Yes	3	2.3	0	0.0	3	100.0	
Electrocardiogram Performed	Yes	133	100.0	130	97.7	3	2.3	
Sinus Rhythm	No	5	3.8	5	100.0	0	0.0	0.729
	Yes	128	96.2	125	97.7	3	2.3	
Type of Conduction Block	None	119	89.5	117	98.3	2	1.7	0.196
	First-Degree AV Block	5	3.8	5	100.0	0	0.0	
	Right Bundle Branch Block (RBBB)	6	4.5	5	83.3	1	16.7	
	Left Bundle Branch Block (LBBB)	3	2.3	3	100.0	0	0.0	
Arrhythmias	None	119	89.5	116	97.5	3	2.5	0.996
	Atrial Fibrillation	3	2.3	3	100.0	0	0.0	
	Ventricular Tachycardia	1	0.8	1	100.0	0	0.0	
	Ventricular Fibrillation	2	1.4	2	100.0	0	0.0	
	Other	8	6	8	100.0	0	0.0	
ST-Segment Elevation	No	72	54.2	70	97.2	2	2.8	0.892
	Yes	61	45.9	60	98.4	1	1.6	
Pathologic Q Wave	No	117	88	114	97.4	3	2.6	0.517
	Yes	16	12	16	100.0	0	0.0	
ProBNP (pg/mL)	Mean (±SD)	773.20	2708.0	772.21	2734.5	815.00	1411.6	0.979 *
Left Ventricular Ejection Fraction (LVEF, %)	Mean (±SD)	51.7	21.1	52.5	20.3	20.2	34.9	0.008 *
Pulmonary Artery Systolic Pressure (PASP, mmHg)	Mean (±SD)	27.42	16.17	28.07	15.78	0.0	0.0	0.003 *
Valvular Heart Disease	None	90	67.7	87	96.7	3	3.3	0.924
	Estenosis aortica	3	2.3	3	100.0	0	0.0	
	Aortic Regurgitation	4	3.0	4	100.0	0	0.0	
	Mitral Stenosis	1	0.8	1	100.0	0	0.0	
	Mitral Regurgitation	17	12.8	17	100.0	0	0.0	
	Other	16	12.0	16	100.0	0	0.0	
Wall Motion Abnormality	None	46	34.6	44	95.7	2	4.3	0.410
	Akinesia	38	28.6	38	100.0	0	0.0	
	Hypokinesia	48	36.1	47	97.9	1	2.1	

bpm: Beats per minute; SD: standard deviation; mmHg: millimeters of mercury; eGFR: estimated glomerular filtration rate; ECG: electrocardiogram; AV: atrioventricular; RBBB: right bundle branch block; LBBB: left bundle branch block; ProBNP: Pro–B-type natriuretic peptide; LVEF: left ventricular ejection fraction; PASP: pulmonary artery systolic pressure. * *p* values calculated from Student’s *t*-test. Values in **bold** indicate statistically significant differences at *p* < 0.05.

**Table 5:** Characteristics and management strategies during hospitalization and at discharge of patients with acute coronary syndrome by outcome.

		Outcome						*p*-Value
		Total		Alive		Deceased		
		n	%	n	%	n	%	
In-Hospital Management								
Hospitalization Days	Mean (±SD)	5	23	6	14	−68	120	N/A
Required ICU Admission	No	42	31.6	40	95.2%	2	4.8%	0.407
	Yes	83	62.4	82	98.8%	1	1.2%	
NYHA Functional Class	I	49	36.8	48	98.0%	1	2.0%	0.932
	II	47	35.3	46	97.9%	1	2.1%	
	III	28	21.1	27	96.4%	1	3.6%	
	IV	9	6.8	9	100.0%	0	0.0%	
Statins	No	3	2.3	0	0.0%	3	100.0%	**<0.001**
	Yes	130	97.7	130	100.0%	0	0.0%	
Beta Blockers	No	58	43.6	55	94.8%	3	5.2%	**0.046**
	Yes	75	56.4	75	100.0%	0	0.0%	
Aspirin (Acetylsalicylic Acid)	No	3	2.3	0	0.0%	3	100.0%	**<0.001**
	Yes	130	97.7	130	100.0%	0	0.0%	
Clopidogrel or Ticagrelor	No	25	18.8	22	88.0%	3	12.0%	**<0.001**
	Yes	108	81.2	108	100.0%	0	0.0%	
Anticoagulants	No	19	14.3	18	94.7%	1	5.3%	0.340
	Yes	114	85.7	112	98.2%	2	1.8%	
Type of Anticoagulant	None	19	14.3	18	94.7%	1	5.3%	**<0.001**
	Enoxaparin	91	68.4	91	100.0%	0	0.0%	
	Rivaroxaban	6	4.5	6	100.0%	0	0.0%	
	Apixaban	10	7.5	10	100.0%	0	0.0%	
	Other	7	5.3	5	71.4%	2	28.6%	
Discharge Management								
NYHA Functional Class	I	70	52.6	67	95.7%	3	4.3%	0.430
	II	57	42.9	57	100.0%	0	0.0%	
	III	4	3.0	4	100.0%	0	0.0%	
	IV	2	1.5	2	100.0%	0	0.0%	
Statins	No	3	2.3	0	0.0%	3	100.0%	**<0.001**
	Yes	130	97.7	130	100.0%	0	0.0%	
Beta Blockers	No	49	36.8	46	93.9%	3	6.1%	**0.022**
	Yes	84	63.2	84	100.0%	0	0.0%	
Aspirin (Acetylsalicylic Acid)	No	3	2.3	0	0.0%	3	100.0%	**<0.001**
	Yes	130	97.7	130	100.0%	0	0.0%	
Clopidogrel or Ticagrelor	No	31	23.3	28	90.3%	3	9.7%	**<0.001**
	Yes	102	76.7	102	100.0%	0	0.0%	
ACE Inhibitor	No	90	67.7	87	96.7%	3	3.3%	0.226
	Yes	43	32.3	43	100.0%	0	0.0%	
Anticoagulants	No	85	63.9	82	96.5%	3	3.5%	0.188
	Yes	48	36.1	48	100.0%	0	0.0%	
Type of Anticoagulant	None	87	65.4	84	96.6%	3	3.4%	0.898
	Enoxaparin	7	5.3	7	100.0%	0	0.0%	
	Warfarin	2	1.5	2	100.0%	0	0.0%	
	Rivaroxaban	20	15.0	20	100.0%	0	0.0%	
	Dabigatran	1	0.8	1	100.0%	0	0.0%	
	Apixaban	16	12.0	16	100.0%	0	0.0%	

SD: Standard deviation; ICU: intensive care unit; NYHA: New York Heart Association; ACE: angiotensin-converting enzyme. Values in **bold** indicate statistically significant differences at *p* < 0.05.

## Data Availability

The data used for this research can be requested from the corresponding author.

## Abbreviations

The following abbreviations are used in this manuscript:

CVDs	cardiovascular diseases
ACS	acute coronary syndrome
DALYs:	disability-adjusted life years
STEMI	ST-segment elevation myocardial infarction
NSTEMI	Non-ST-segment elevation myocardial infarction
WHO	World Health Organization
IHD	ischemic heart disease
